# EZH2 regulates pancreatic cancer cells through E2F1, GLI1, CDK3, and Mcm4

**DOI:** 10.1186/s41065-023-00280-1

**Published:** 2023-05-17

**Authors:** Hongfeng Li, Hailong Wang, Yunlong Cui, Wenhua Jiang, Hongjie Zhan, Lixia Feng, Mingyou Gao, Kuo Zhao, Limeng Zhang, Xiaojing Xie, Ning Zhao, Ying Li, Pengfei Liu

**Affiliations:** 1 Department of Clinical Laboratory, Tianjin Academy of Traditional Chinese Medicine Affiliated Hospital, Tianjin, 300120 China; 2 Department of Oncology, Tianjin Academy of Traditional Chinese Medicine Affiliated Hospital, No. 354 Beima Road, Hongqiao District, Tianjin, 300120 China; 3 Department of Hepatobiliary Oncology, National Clinical Research Center for Cancer, Key Laboratory of Cancer Prevention and Therapy of Tianjin, Tianjin’s Clinical Research Center for Cancer, Tianjin Medical University Cancer Institute and Hospital, Tianjin, 300060 China; 4 Department of Radiotherapy, The Second Hospital of Tianjin Medical University, Tianjin, 300211 China; 5 Department of Gastric Cancer, National Clinical Research Center of Cancer, Key Laboratory of Cancer Prevention and Therapy of Tianjin, Tianjin Medical University Cancer Institute and Hospital, Tianjin, 300060 China; 6 Department of Nursing, National Clinical Research Center of Cancer, Key Laboratory of Cancer Prevention and Therapy of Tianjin, Tianjin Medical University Cancer Institute and Hospital, Konggang Hospital, Tianjin, 300300 China; 7 Department of Oncology, National Clinical Research Center for Cancer, Key Laboratory of Cancer Prevention and Therapy of Tianjin, Tianjin Medical University Cancer Institute and Hospital, Tianjin, 300060 China; 8 Department of Medical Oncology, The Fourth Hospital of Hebei Medical University, No. 12 Health Road, Shijiazhuang, 050000 Hebei China

**Keywords:** PC, *EZH2*, Proliferation, Migration, Transcriptome

## Abstract

Pancreatic cancer (PC) is one of the most common malignant tumors in digestive tract. To explore the role of epigenetic factor *EZH2* in the malignant proliferation of PC, so as to provide effective medical help in PC. Sixty paraffin sections of PC were collected and the expression of *EZH2* in PC tissues was detected by immunohistochemical assay. Three normal pancreas tissue samples were used as controls. The regulation of *EZH2* gene on proliferation and migration of normal pancreatic cell and PC cell were determined by MTS, colony forming, Ki-67 antibody, scratch and Transwell assays. Through differential gene annotation and differential gene signaling pathway analysis, differentially expressed genes related to cell proliferation were selected and verified by RT-qPCR. *EZH2* is mainly expressed in the nuclei of pancreatic tumor cells, but not in normal pancreatic cells. The results of cell function experiments showed that *EZH2* overexpression could enhance the proliferation and migration ability of PC cell BXPC-3. Cell proliferation ability increased by 38% compared to the control group. *EZH2* knockdown resulted in reduced proliferation and migration ability of cells. Compared with control, proliferation ability of cells reduced by 16%-40%. The results of bioinformatics analysis of transcriptome data and RT-qPCR demonstrated that *EZH2* could regulate the expression of *E2F1*, *GLI1*, *CDK3* and *Mcm4* in normal and PC cells. The results revealed that *EZH2* might regulate the proliferation of normal pancreatic cell and PC cell through *E2F1*, *GLI1*, *CDK3* and *Mcm4*.

**Supplementary Information:**

The online version contains supplementary material available at 10.1186/s41065-023-00280-1.

## Introduction

PC is one of the most common malignant tumors of the digestive tract, and its morbidity and mortality have increased significantly in recent years. In 2020, 57,600 new PC cases and 47,050 new deaths are projected to occur in the United States [31]. The early diagnosis of PC is very difficult since it is prone to metastasize and has no obvious clinical symptoms. About 75% of patients have been diagnosed with stage III or IV. Most patients die within one year after diagnosis of PC, and the 5-year survival rate is less than 1%, which is one of the malignant tumors with the worst prognosis [12]. PC is a complex disease associated with multiple genes. Many studies have found that the abnormal expression or activation of multiple signaling pathways (e.g. Insulin-Like Growth Factor (IGF) pathway), growth factors (e.g. Vascular Endothelial Growth Factor (VEGF)), oncogenes (e.g. K-ras) and suppressor genes (e.g. DCC) play a role in the occurrence and development of PC [3, 6, 29].

For PC, there has been a lack of effective adjuvant therapy. The malignant degree of PC cells is extremely high, and their growth rate is too fast. Moreover, the cancer cells have little sensitivity to radiotherapy and chemotherapy, resulting in poor curative effects [9, 27]. At present, there is a lack of specific targeted therapy for PC [34]. Radical surgical resection is the only chance for patients with PC to have a longer survival [32]. However, most patients are already at advanced stage when diagnosed, causing about 85% of patients to lose opportunity for radical surgery due to local important blood vessel invasion or distant metastasis. Therefore, in-depth exploration of the pathogenesis of PC to search biomarkers for early diagnosis, prognosis, and therapeutic targets is the key to improve the diagnosis and treatment of PC [23].

Enhancer of zeste homolog 2 (*EZH2*) is located on human chromosome 7q35 andhighly conserved, with three conserved sequences at the N-terminus and an evolutionarily highly conserved SET domain at the C-terminus [4]. *EZH2* is a catalytic subunit of PRC2, one of the core proteins of the epigenetic modification system polycomb group (PCG). *EZH2* protein is a trimethylase of lysine 27 of nucleosome histone H3 and is an important transcriptional regulatory factor [5]. The transcriptional repression mediated by *EZH2* relies on the complete SET domain. If this region is lost, it does not produce a suppressive phenotype, and degenerates genes in some cases. A large number of studies have indicated that the expression of *EZH2* in cancer tissues is abnormally higher than that in normal tissues in a variety of cancers, such as pancreatic [11], prostate [17] and breast cancer [26]. *EZH2* gene plays an important role in the proliferation, migration, invasion, apoptosis and cycle of cancer cells by regulating several important signaling pathways, such as Wnt, RAS, NF-κB and NOTCH [36]. It is considered to be a very valuable drug target [18]. The main research goal of this study is to reveal the expression of *EZH2* in PC and its effect on the biology function of PC cell, and to preliminarily explore the molecular mechanism of its action, so as to provide theoretical reference and experimental basis for the development of new drug targets of PC.

## Materials and methods

### Patients and sample collection

Our study collected the paraffin sections of 60 patients with PC who underwent surgical resection at Tianjin Medical University Cancer Institute and Hospital from August 2005 to November 2013. All patients were not given radiotherapy and chemotherapy before surgery and were confirmed to be PC by pathological examination. Three other normal pancreatic tissue samples were selected as controls. Written consent was signed by each subject that enrolled in this study.

### Immunohistochemistry

The paraffin sections of PC and normal tissues were isolated and used for immunohistochemistry analysis according to the frequently-used protocol. In short, the samples were treated with conventional dewaxing to water, performed with high pressure repair antigen. The sections were incubated with a monoclonal mouse anti-*EZH2* antibody (Sigma-Aldrich). After washing with PBS, the sections were incubated with secondary antibody for 20 min at 37 °C. The slides were stained with diaminobenzidine (DAB) for longer than 20 s and less than 7 min, then washed with water. Photographs were observed under an fluorescence microscope.

### Cells and plasmids

The human PC cells BXPC-3 and the human normal pancreatic cells HPDE6-C7 were purchased from the Cell Resource Center of Shanghai Academy of Sciences, CAS and preserved by our laboratory. The lentiviral expression vector PCDH-CMV-MCS-EFl-Puro (CD510B-1) and packaging plasmids VSV-G, RSV-REV and pMDL were stored by this laboratory. *EZH2* shRNA interference plasmid pGIPZ-V2LTHS and lentiviral expression vector pGIPZ were purchased from BD Biosciences (USA) and stored by this laboratory (Supplementary Table 1). The overexpression and set domain deletion plasmids of *EZH2* gene were constructed by our laboratory. The plasmids were verified by PCR method and plasmid sequencing technology.

### Construction of stable transfected cells model of EZH2

Lentiviral transfection was used to transfect the above successfully verified plasmids into HPDE6-C7 and BXPC-3, respectively. The main experimental process was to seed cells into 6-well plates and conduct cell infection experiments when cell confluence reached 50%-70%. The reaction system was 400 μL virus and 1600 μL fresh medium. 8–10 μg/mL polybrene was added, and centrifuged at 2500 rpm and 37 °C for 30 min. After incubated for 24 h, the medium in the infected cells was changed. The expression of *EZH2* was detected by real-time (RT)-qPCR (primer sequences were shown in Supplementary Table 2) and Western blot, so as to verify whether the stable cells of *EZH2* were successfully constructed.

### RT-qPCR analysis

Cells in log phase were collected, total RNA was extracted by Trizol method, and then cDNA was synthesized by reverse transcription for RT-qPCR analysis (Genewiz, China). In this study, RT-qPCR primers were designed by ourselves and synthesized commercially by Genewiz, China. The primers were listed in Supplementary Table 2. β-actin was used as inner control.

### Cell proliferation assay

#### Cell growth assay

Cells were plated in 96-well plates, at a density of 4 *103 cells per well, and cultured overnight. The number of viable cells was quantified every 24 h for 5 days by counting cells using hemocytometer.

#### MTS assay

The3-(4,5-dimethylthiazol-2-yl)-5-(3-carboxymethoxyphenyl)-2-(4-sulfophenyl)-2H-tetrazoliu,inner salt (MTS) assay was used to measure the effect of *EZH2* on the proliferation of the stably transfected cells. The different of *EZH2* stable transfected cells models in the logarithmic growth phase were collected, and the density of single cell suspension was adjusted to 2–3 × 10^5^ cells/mL. 200 μL single cell suspension was added to the well of the 96-well plate, and 6 parallel wells were set for each group, the wells with only medium were set as blank control group. Then the cells were incubated in 5% CO2 at 37 °C for 48 h. The basal medium: MTS: PUS was thoroughly mixed at a ratio of 100: 20: 5 to prepare the mixture. Each well was added with 120 μL mixture and continued to incubate in 5% CO2 at 37 °C for 0.5 h. The optical density (OD) of each well at 490 nm was measured by microplate reader. The reference wavelength of 620 nm was used to subtract the background value caused by excessive cell debris and other non-specific OD values.

#### Colony forming assay

The cell concentration was adjusted to 1000 cells/mL, seeded into 6-well plate, and cultured in 5% CO2 at 37 °C for 7–10 days. When clones were visible with the naked eyes, they were fixed with methanol for 15 min, and then air-dried after discarding the methanol. Giemsa staining solution was used for staining for 10–15 min, and photos were taken and recorded.

#### Ki-67 antibody test

The expression of Ki-67 was detected using Ki-67 detection kit (BD Biosciences, MD USA), according to the manufacturer’s protocol.

### Cell migration assay

#### Transwell test

100 μL cells with a concentration of 5 × 10^5^ cells/mL were added to the transwell chamber and routinely cultured for 12 h at 37°Cand 5% CO2. The medium was removed and 800 μL methanol was added to fix the cells for 30 min. Then methanol was discarded and the cells were placed in the culture wells with 500 μL crystal violet dye and dyed for 15 min. The cells that permeated the filter were counted.

#### Scratch test

Each group of cells were resuspended and seeded onto a 6-well plate. After 24 h, a horizontal ‘scratch’ was made using a pipette tip in the cell monolayer. The cells were washed with PBS for 3 times, then medium without serum was added and the cells were cultured at 37 °C and 5% CO2. Cell migration into the scratched area was observed and imaged at 0, 24 and 48 h, respectively.

### Transcriptome sequencing and bioinformatics analysis

*EZH2* knockout stably transfected cell line *EZH2*-sh17509-BXPC-3 and empty control pGIPZ-BXPC-3 cells were cultured to the concentration of 1 × 10^7^ cells/mL. The qualified cell samples were directly sent to Novogene Biotechnology Co, Ltd (Beijing, China) for transcriptome sequencing. The amount of sequencing data was 5 Gb per sample. We directly performed bioinformatics analysis based on clean reads provided by sequencing company.

### Bioinformatics analysis

Clean reads were compared with human reference genome sequences (hg19.fa) and control-shRNA sequence to obtain the expression abundance of corresponding genes in samples. According to the abundance of genes expression in different samples, the differentially expressed genes (DEGs) were searched. The overall comparison was made according to the functional classification of the DEGs on the Gene Ontology (GO). The biological pathways of enriched DEGs were analyzed on Kyoto Encyclopedia of Genes and Genomes (KEGG) to explore their pathways and key nodes.

### RT-qPCR validation analysis

RT-qPCR was used to detect the expression level of screened DEGs. The test method is the same as above.

### Statistical analysis

The results were presented as mean ± standard deviation (SD). SPSS 21 software was used for statistical analysis. Comparison between two groups were performed by *t* test (data was found to be normal distribution). One-way ANOVA was used for comparison between more than two groups (The variance of different groups is the same). The standard of this study was *P* < 0.05 with statistical difference.

## Results

### The expression level of EZH2 in clinical samples of PC

**Fig. 1:**
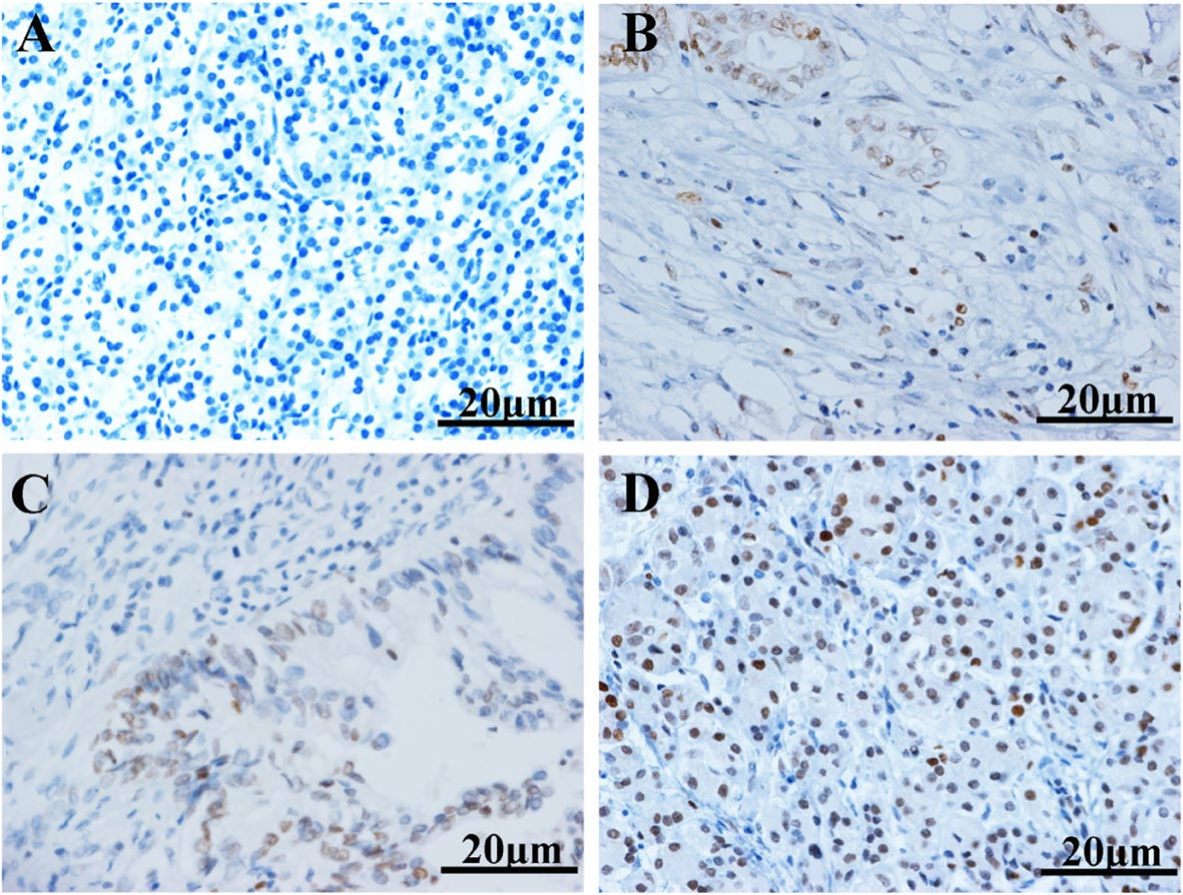
The immunohistochemical results of EZH2 was different in different clinical samples. **A** Normal pancreatic tissue; **B** Pancreatic ductal carcinoma; **C** Solid Pseudopapillary Neoplasm; D) Neuroendocrine tumor. The pale yellow to brownish yellow granules indicated EZH2 expression, and blue indicates no expression

The results of immunohistochemical experiments based on clinical samples showed that *EZH2* was mainly expressed in the nuclei of tumor cells, showing pale yellow to brownish yellow granules (Fig. 1A). While normal pancreatic tissue was blue, indicating that no *EZH2* expression was observed (Fig. 1B-D).

**Fig. 2:**
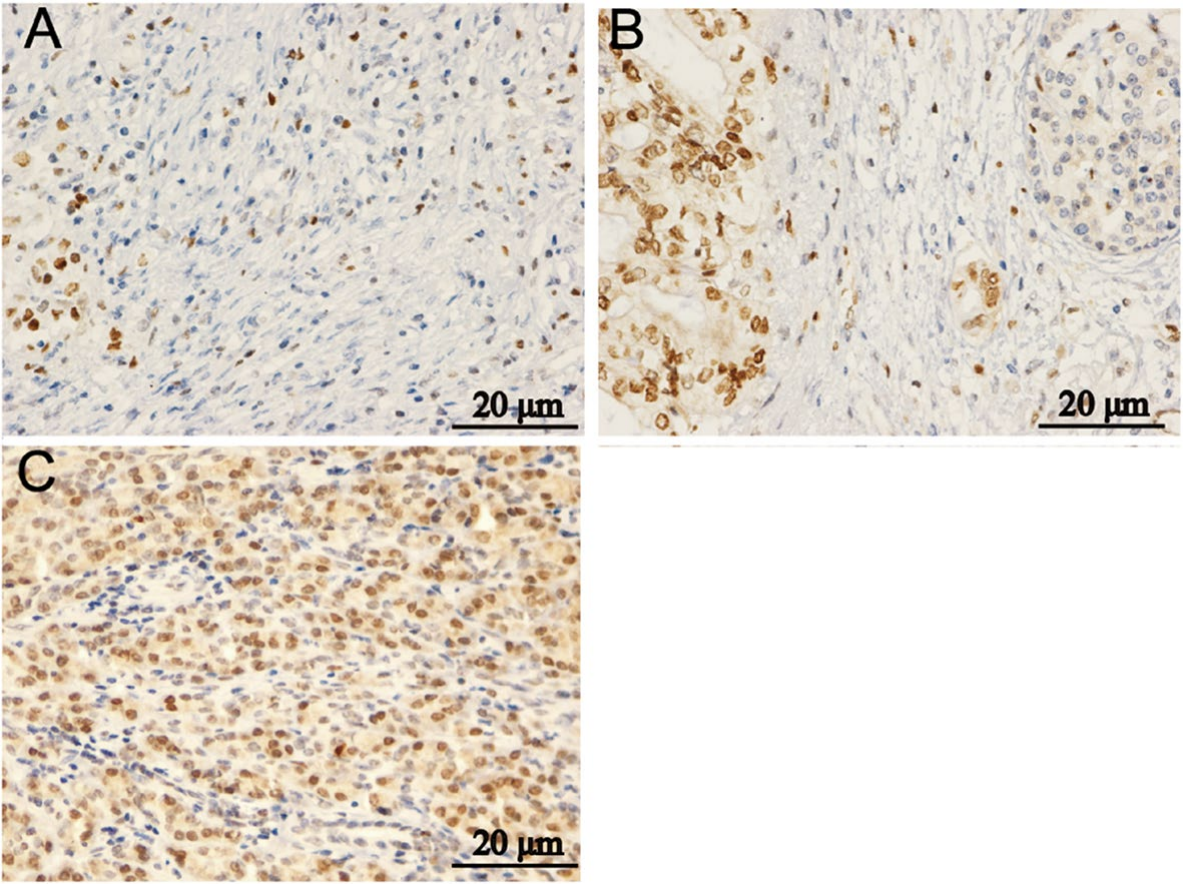
EZH2 was different in PC tissue samples with different pathological grades. **A** highly differentiated; **B** medium differentiated; **C** low differentiated

In order to analyze the correlation between the expression level of *EZH2* and the malignant degree of PC, we classified these PC cases into high differentiation, moderate differentiation and low differentiation according to the degree of cell differentiation, cell heteromorphism, and mitosis. The results showed that *EZH2* was expressed less in highly differentiated PC (Fig. 2A), more in medium-differentiated PC (Fig. 2B), and most in low-differentiated PC (Fig. 2C). It can be seen that the expression level of EZH2 is positively correlated with the malignancy of PC.

### Establishment of EZH2 overexpression and knockdown stably transfected cell line

We constructed 7 *EZH2* stable transfected cells model of normal pancreatic cells HPDE6-C7 and PC cells BXPC-3, respectively (Supplementary Table 3). RT-qPCR and Western blot experiments were applied to detect the expression level of *EZH2* in each group of cell lines.

**Fig. 3:**
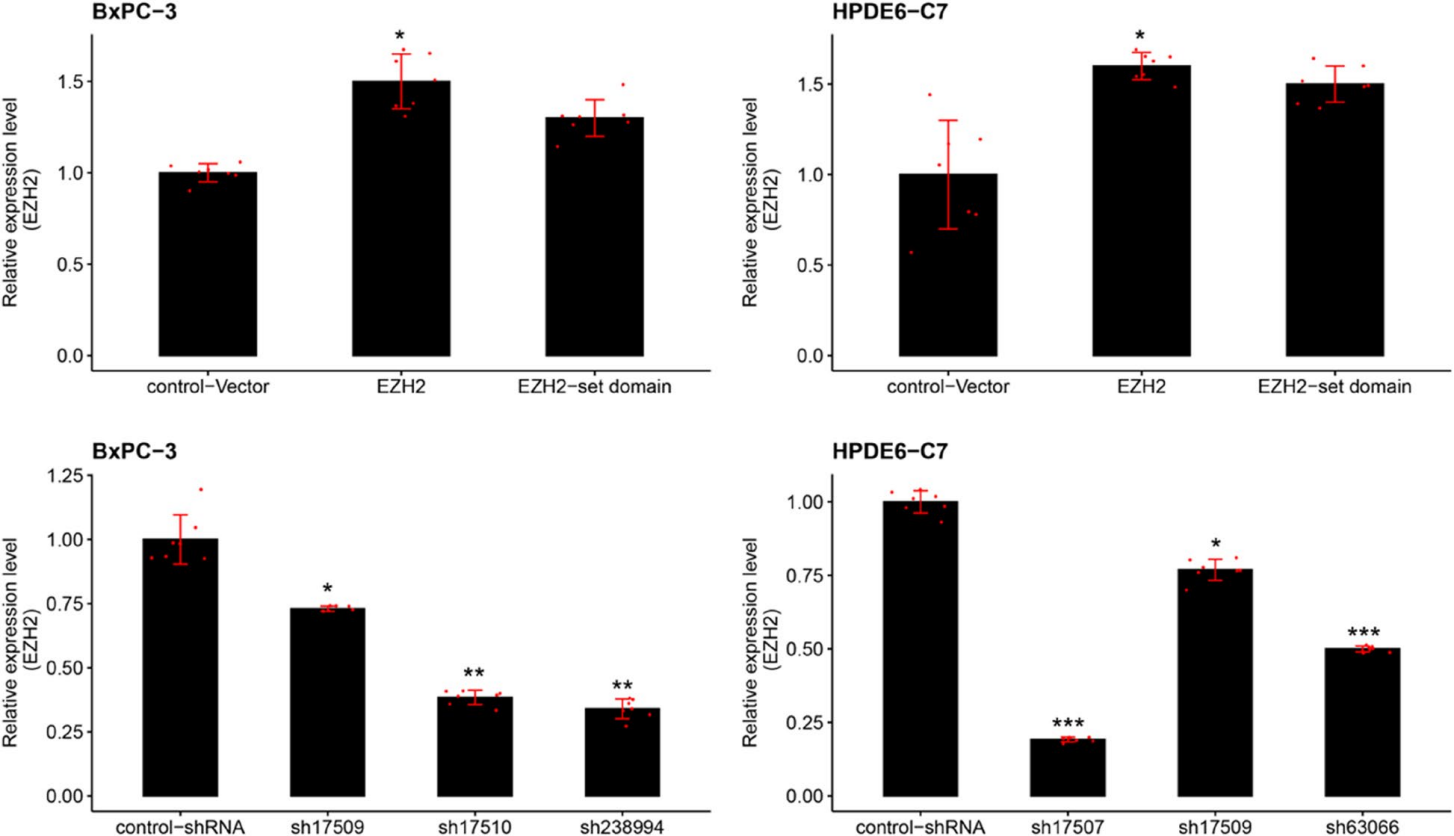
RT-qPCR detected the expression level of EZH2 mRNA in the establishment of EZH2 overexpression and knockdown cell models. **A** and **C** showed the BXPC-3 cells, **B** and **D** showed the HPDE6-C7 cells. The internal reference target was β-actin gene. The value of 2-∆∆Ct represents the fold change in the expression of the target gene relative to the control group, with a value of 1 indicating no change in gene expression. *,**,*** represented significant differences between the different of *EZH2* stable transfected cells models, *P* < 0.05, *P* < 0.01, and *P* < 0.001, respectively

RT-qPCR results showed that in *EZH2* overexpression and *EZH2* set domain deletion BXPC-3 and HPDE6-C7 cells, *EZH2* mRNA expression was up-regulated and reached 150% and 160%, 130% and 150% of the control-vector group, respectively (Fig. 3A-B). In the *EZH2* knockdown cells, the expression level of *EZH2* mRNA was dramatically lower than that of the control-shRNA group (*P* < 0.05), as shown in Fig. 3C and D.

At the protein level, the expression levels of EZH2 were higher in EZH2 overexpression and *EZH2* set domain deletion BXPC-3 cells (Supplementary Fig. 1A). The corresponding grayscale analysis results demonstrated that the EZH2 protein expression levels of this two groups were 270% and 280% of the control-vector group, respectively (Supplementary Fig. 1C). The same trend was seen in normal pancreatic cells HPDE6-C7, as shown in Supplementary Fig. 1B and D. The expression level of EZH2 protein in *EZH2* knockdown groups was observably lower than that in the control-shRNA group (*P* < 0.05, Supplementary Fig. 1A, B, E and F).

The results of RT-qPCR and Western blot indicated that the *EZH2* overexpression and knockdown stably transfected cell lines were successfully constructed.

### Effects of EZH2 on the biological characteristics of cells

#### Effects on proliferation ability

**Fig. 4:**
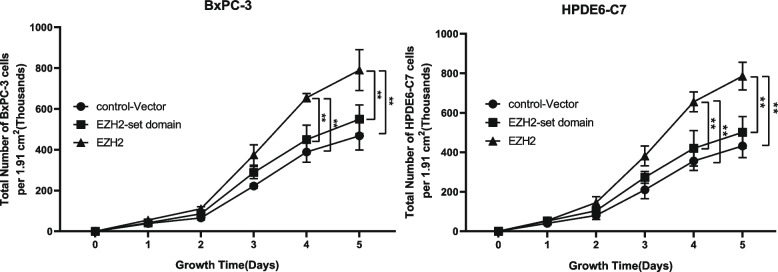
Cell growth experiment demonstrated cell proliferation rate of BXPC-3 cells, and HPDE6-C7 cells in different groups. *,**,*** represented significant differences between the different of EZH2 stable transfected cells models, *P* < 0.05, *P* < 0.01, and *P* < 0.001, respectively

**Fig. 5:**
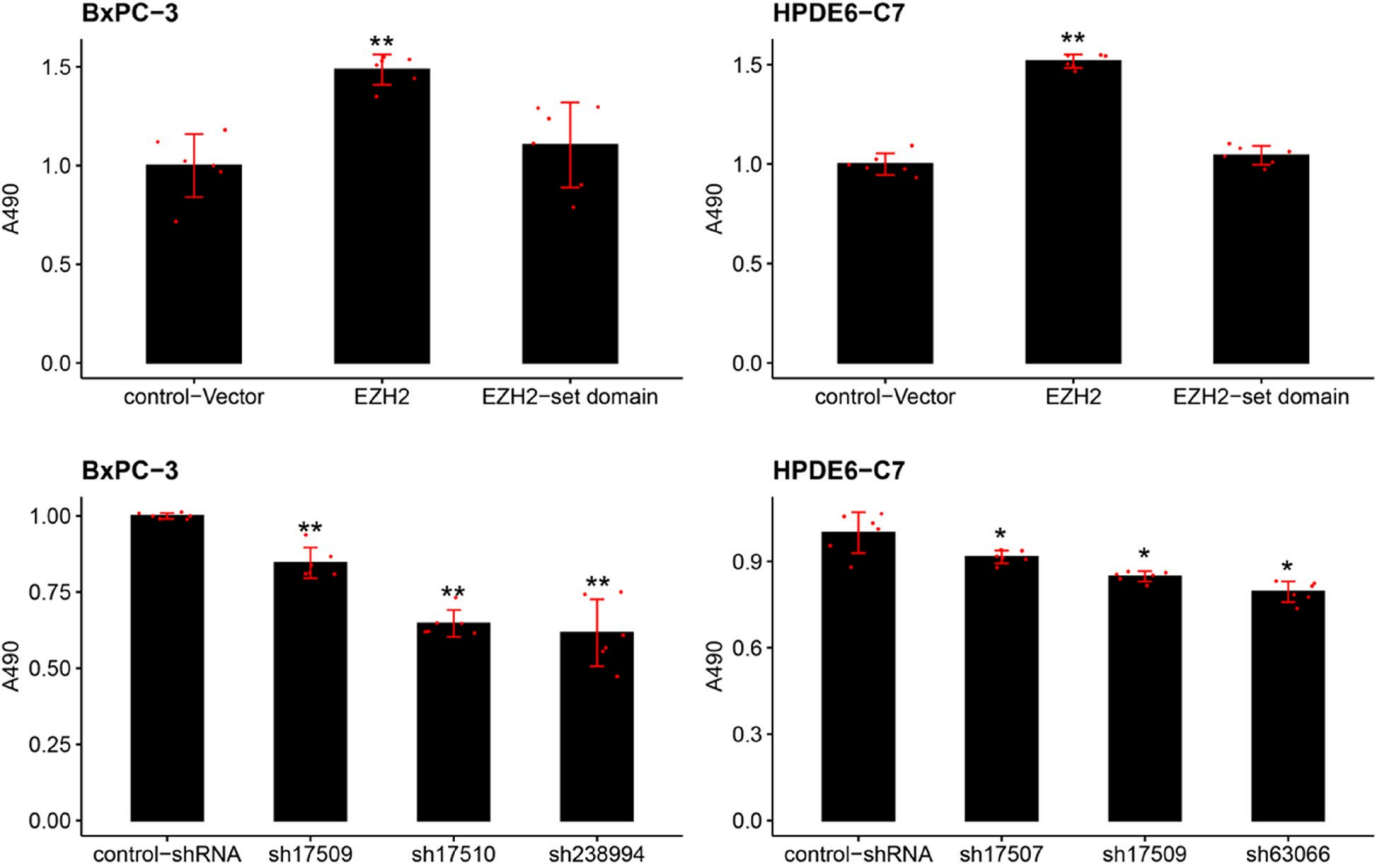
MTS researched the cell proliferation ability in the established EZH2 overexpression and knockdown cell models. **A** and **C** showed the BXPC-3 cells, **B** and **D** showed the HPDE6-C7 cells. MTS results are presented as a percentage of control, where the absorbance values of the treatment group are divided by the absorbance values of the control group and multiplied by 100 to give a percentage value. *,**,*** represented significant differences between the different of *EZH2* stable transfected cells models, *P* < 0.05, *P* < 0.01, and *P* < 0.001, respectively

By using simple cell growth experiment, it showed that two types of cancer cells had higher proliferation rate in EZH2 transfected groups when compared to control groups (Fig. 4). The results of MTS experiments revealed that OD_490_ in *EZH2* overexpression cell lines were higher than that in control-vector, and the difference was statistically significant, indicating that the cell proliferation ability was markedly enhanced (Fig. 5A-B). The OD_490_ value of *EZH2* set domain deletion cell was slightly higher than that of the control-vector group, but the difference was not significant, and the cell proliferation ability was slightly increased (Fig. 5A-B). Compared with control-vector group, the OD_490_ values of each *EZH2* knockdown cell lines were lower, and the difference was statistically significant. (Fig. 5C-D).

In the colony formation experiments, there were a certain number of visible cell clones in the stably transfected cell lines of each group. The number of colonies and the size of individual colony formed by *EZH2* overexpression cell lines were meaningfully higher than those of the *EZH2* set domain deletion and control-vector groups (Supplementary Fig. 2A-B). *EZH2* knockdown resulted in smaller colonies and lighter staining (Supplementary Fig. 2C-D).

Ki-67 antibody assay results proved that the percentage of proliferating cells of *EZH2* overexpression, *EZH2* set domain deletion and control-vector BXPC-3 cells were 75.6% and 58.6% and 60%, respectively (Supplementary Fig. 3A). In each *EZH2* knockdown and control-shRNA BXPC-3 cells, the percentage of proliferating cells were 42.9%, 35.4%, 34%, and 67.5%, separately (Supplementary Fig. 3C). The percentage of proliferating cells of HPDE6-C7 cells in each groups showed a similar trend to that of BXPC-3 cells (Supplementary Fig. 3B and D).

The results of MTS, colony formation and Ki-67 antibody experiments made clear that *EZH2* overexpression promoted cell proliferation, *EZH2* knockdown inhibited cell proliferation, and *EZH2* set domain deletion did not significantly affect cell proliferation..

#### Effects on migration ability

**Fig. 6:**
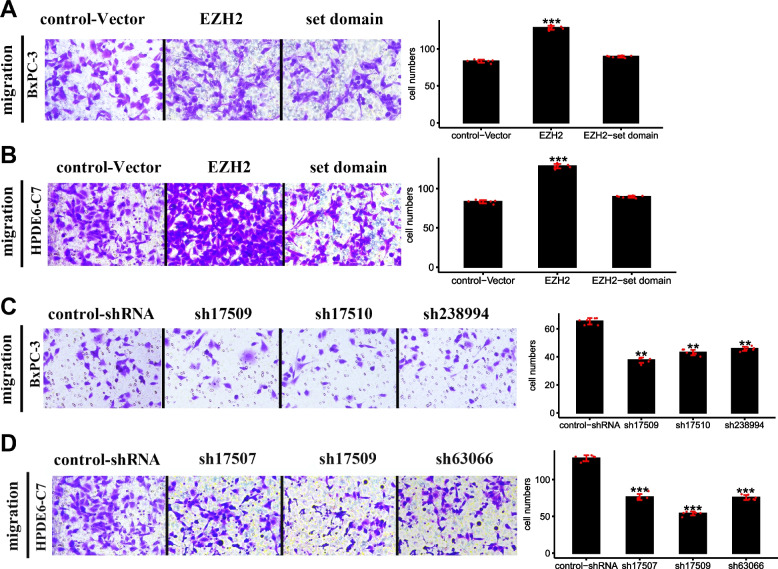
Transwell assay detected the cell migration in different cell models. **A** and **C** showed the BXPC-3 cells, **B** and **D** showed the HPDE6-C7 cells. Representative figures for migrated cells per field were shown. Then the term "cell numbers" was annotated as the total cells per well. *,**,*** represented significant differences between the different of *EZH2* stable transfected cells models, *P* < 0.05, *P* < 0.01, and *P* < 0.001, respectively

Transwell results showed that the number of cell migration of *EZH2* overexpression, *EZH2* set domain deletion, and control-vector BXPC-3 cell model were 125, 86, and 81 (Fig. 6A). Each *EZH2* knockdown and control-shRNA cell migration numbers were 36, 42, 44, and 64 (Fig. 6C). In *EZH2* overexpression and knockdown HPDE6-C7 cell models, the trend of cell migration number was similar to that of BXPC-3 cell (Fig. 6B and D).

In the scratch test, the distance between the two scratch lines of the *EZH2* overexpression cells was prominently narrower than the *EZH2* set domain deletion and the control-vector cells (Supplementary Fig. 4A and C, Fig. 6A and C). Each *EZH2* knockdown groups had striking wider distance between the two scratch lines than control-shRNA cells (Supplementary Fig. 4B and D, B and D).

The results of Transwell and scratch tests proved that *EZH2* overexpression improved cell migration ability, *EZH2* knockdown weakened cell migration ability, and *EZH2* set domain deletion had little effect on cell migration ability.

### Transcriptome studies on EZH2 knockdown PC cells

**Fig. 7:**
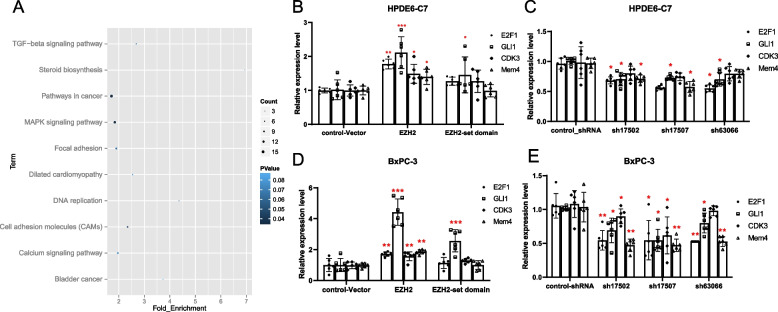
**A** The top 10 KEGG pathways enriched for DEGs (P-value from small to large). Count represented the number of enriched DEGs, *P*-value represents the clustering DEGs analysis. **B**-**E** The mRNA expression level of E2F1, GLI1, CDK3 and Mcm4 were determined by RT-qPCR. The internal reference target was β-actin gene. The value of 2-∆∆Ct represents the fold change in the expression of the target gene relative to the control group, with a value of 1 indicating no change in gene expression. *,**,*** represented significant differences between the different of *EZH2* stable transfected cells models, *P* < 0.05, *P* < 0.01, and *P* < 0.001, respectively

593 DEGs were detected in *EZH2* knockdown cells *EZH2*_sh17509. Supplementary Table 4 listed the top 30 DEGs screened by log2FC. Through the KEGG signal pathway enrichment analysis of DEGs, a total of 76 signaling pathways were enriched. According to the order of *P*-Value from small to large, Fig. 7A showed the top 10 signaling pathways. Among them, only Pathways in cancer, MAPK signaling pathway and Cell adhesion molecules (CAMs) had *P*-values less than 0.05, and the number of involved DEGs was 15, 13, and 8, indicating that these DEGs might play an important role in the occurrence and development of PC. By referring to related literatures and databases, E2F1, GLI1, CDK3, and Mcm4, as candidate target genes, were found to be closely related to the occurrence of cancers, which was very helpful to clarify the mechanism of *EZH2* in PC. E2F1, GLI1, CDK3 and Mcm4 were selected as the targeted genes and subsequently verified.

The results of RT-qPCR experiment demonstrated that *EZH2* overexpression promoted the expression of E2F1, GLI1, CDK3 and Mcm4, and when the set-domain was deleted, the promotion effect disappeared. *EZH2* knockdown could inhibit the expression of these genes (Fig. 7B-E). These results were consistent with transcriptome sequencing results.

## Discussion

We have found that abnormal expression of *EZH2* can be detected in various cancer tissues. During tumorigenesis and development, the expression level of *EZH2* was continuously increasing. Tang et al. indicated that EZH2 staining was positive in cholangiocarcinoma specimens, while EZH2 staining was negative in normal biliary epithelial tissue [35]. In this study, the expression of *EZH2* in normal pancreatic and PC tissues was studied by immunohistochemistry. We found that *EZH2* was expressed in the nuclei of tumor cells, but not in normal pancreatic tissues. Furthermore, the expression levels of *EZH2* were also different in PC tissues with different histological grades, that is, less in highly differentiated PC, more in moderately differentiated PC, and most in highly differentiated PC. This is consistent with the findings of *EZH2* in a variety of other tumors, including liver [10], lung [37] and breast cancer [28]. Because the different histological grades reflect the malignant degree of PC, that is to say, the malignant grade of the highly differentiated tumor is low, while the malignant grade of the poorly differentiated tumor is high. Therefore, the results of this study had verified a certain relationship between *EZH2* expression and the occurrence and development of PC, and also we indicated that *EZH2* expression can assist with the diagnosis of malignant degree of PC.

The biological behavior characteristics of tumor cells are often different from those of normal cells, and these features allow tumor cells to maintain the growth advantage and fight and evade attack from the body's immune system. Their unique biological behavior characteristics include unlimited proliferation, loss of contact inhibition, active migration and invasion, etc. [2]. Among them, cell proliferation and migration are important factors affecting tumor recurrence and prognosis. Many studies have shown that *EZH2* is related to the malignant biological characteristics of tumors, such as tumor malignant proliferation, cycle, apoptosis and migration. Liu et al. found that *EZH2* promoted migration and invasion of renal cell carcinoma (RCC) cell lines, and might contribute to RCC progression and was a potential therapeutic target for advanced RCC [21]. Eskander et al. created stable *EZH2* knockdown endometrial cancer cell lines and proved that RNA interference of *EZH2* expression significantly decreased cell proliferation, migration, and invasion [7]. Li et al. demonstrate that the silence of *EZH2* inhibited cell growth and the cell cycle, and promoted the progression of apoptosis of prostate cancer stem cells (PCSC), and *EZH2* was essential for PCSC growth [17]. Therefore, it can be seen that understanding the effect of *EZH2* on the biological characteristics of normal pancreatic cells and PC cells is an important basis for studying the mechanism of EZH2 in the development of PC. In this study, based on the results of MTS, Ki-67 and colony formation experiments we observed that *EZH2* promoted the proliferation of normal pancreatic cells and PC cells. Through scratch test and Transwell experiment, we found that *EZH2* overexpression could enhance the migration ability of PC cells. Our results provided an important basis for further research on the mechanism of *EZH2* in PC.

The transcriptome is a collection of all products transcribed from a specific tissue or cell at a certain developmental stage or functional state. Transcriptome research can study gene function and gene structure as a whole, and reveal the molecular mechanism of specific biological process and disease occurrence process [25]. This technique is increasingly applied to the study of tumor function and related mechanisms. The mechanism of *EZH2* in PC is still unclear. We preliminarily explored the mechanism of *EZH2* in PC by transcriptome analysis, and found that *EZH2* may regulate the expression of E2F1, GLI1, CDK3 and Mcm4.

E2 promoter binding factor 1 (E2F1) belongs to the transcription factor family named E2Fs, which is an intracellular transcription factor and the first member of the E2F family to be identified [39]. As an important regulator of cell cycle, E2F1participates in regulating the cell transition from G0/G1 phase to S phase. E2F1 is also involved in processes such as cell cycle arrest, apoptosis and cell differentiation [24]. Recent studies have found that E2F1 abnormally expresses in a variety of malignant tumors, such as breast cancer [13], prostate cancer [20], ovarian cancer [8], suggesting that it may be an important pathogenic factor in tumorigenesis and development. Glioma-associated oncogene homolog 1 (*GLI1*) is a gene that functions as a transcription factor and acts as a transcriptional activator. It is a component and the key signaling molecule of the classic Hedgehog signaling pathway. *GLI1* is a marker gene activated by the Sonic Hedgehog (Shh) signaling pathway, which can activate and accelerate the transcription and translation of downstream genes [41]. As a result, the abnormal proliferation, differentiation and metastasis of cells lead to the occurrence of tumors. Studies have indicated that *GLI1* abnormally expresses in breast cancer [33], colorectal cancer [1], esophageal cancer [40] and other malignant tumors, and can also regulate the activity of other transcription factors to stimulate the proliferation, differentiation, and transformation of tumor cell. CDKs (cyclin-dependent kinases) are the core of the cell cycle regulation mechanism, and necessary condition for the initiation and progress of various molecular events in the cell cycle when combination with the corresponding cyclin. Among CDKs, CDK3, combined with the corresponding cyclin, is involved in regulating cell G1/S transition and promoting cell G0 phase exit, which is the rate-limiting step in the cell cycle process [30]. *CDK3* is found to be expressed in a variety of human tissues, and the activity was extremely low in most normal tissues or cells. In colon cancer [22], nasopharyngeal cancer [38] and other tumor tissues, *CDK3* had been found to have an unusually high expression rate, playing an important role in cell proliferation and malignant carcinogenesis. The Mcm (minichromosome maintenance) family is a class of highly conserved proteins that are involved in DNA replication, transcription, and repair. The Mcm family member Mcm4 is essential for initiating eukaryotic genomic DNA replication, and is a key component of the eukaryotic cell DNA pre-replication complex [15]. Mcm4 abnormally expresses in tumors such as gastric cancer [19], esophageal cancer [14] and breast cancer [16], and is a special marker of proliferating cells. Taken together, *E2F1, GLI1, CDK3* and *Mcm4* all have the effect of promoting cell proliferation. In this current study, our transcriptome bioinformatics analysis revealed that *E2F1, GLI1, CDK3*, and *Mcm4* are important DEGs in EZH2 knockdown BXPC-3 cells. In addition, our RT-qPCR also further demonstrated that *EZH2* could regulate the expression of *E2F1, GLI1, CDK3* and *Mcm4*. It is suggested that the regulation of *EZH2* in PC may be related to *E2F1, GLI1, CDK3* and *Mcm4*.

## Conclusion

In short, *EZH2* was mainly expressed in the nuclei of PC tumor cells. Moreover, *EZH2* might regulate the proliferation cells through *E2F1, GLI1, CDK3*, and *Mcm4*. This study preliminarily explained the mechanism of *EZH2* in the malignant proliferation of PC. In the future, *EZH2* may be widely used as a diagnostic marker and drug target for PC in clinical practice.

## Supplementary Information

**Additional file 1: Supplementary Table 1. **EZH2 shRNA sequences.** Supplementary Table 2. **RT-qPCR primer sequences.** Supplementary Table 3. **The list of EZH2 overexpression and knockdown stably transfected cell line.** Supplementary Table 4. **The list of Top 30 DEGs.** Supplementary Figure 1. **Western blot detected the expression level of EZH2 protein in the establishment of EZH2 overexpression and knockdown cell models. A, C and E showed the BXPC-3 cells, B, D and F showed the HPDE6-C7 cells. The internal reference target was β-actin gene. *,**,*** represented significant differences between the different of EZH2 stable transfected cells models, *P* < 0.05, *P* < 0.01, and *P* < 0.001, respectively.** Supplementary Figure 2. **Effect of EZH2 on cell proliferation detected by colony formation experiment. A and C showed the BXPC-3 cells, B and D showed the HPDE6-C7 cells.** Supplementary Figure 3. **Gating strategy for living/dead cells exclusion by staining 7AAD.** Supplementary Figure 4. **Ki-67 antibody experiments tested the effect of EZH2 on cell proliferation. A and C showed the BXPC-3 cells, B and D showed the HPDE6-C7 cells. *,**,*** represented significant differences between the different of EZH2 stable transfected cells models, *P* < 0.05, *P* < 0.01, and *P* < 0.001, respectively.** Supplementary Figure 5. **The influence of EZH2 on cell migration in HPDE6-C7 cell lines was detected by scratch test. *,**,*** represented significant differences between the different of EZH2 stable transfected cells models, *P* < 0.05, *P* < 0.01, and *P* < 0.001, respectively.** Supplementary Figure 6. **Scratch test detected the cell migration of EZH2 overexpression and knockdown BXPC-3 cell models. *,**,*** represented significant differences between the different of EZH2 stable transfected cells models, *P* < 0.05, *P* < 0.01, and *P* < 0.001, respectively. 

## Data Availability

The dataset used and/or analyzed during the current study are available from the corresponding author on reasonable request.
