# Supplementary material for: EZH2 regulates pancreatic cancer cells through E2F1, GLI1, CDK3, and Mcm4
Source: Hereditas. 2023 May 17;160:23. doi: 10.1186/s41065-023-00280-1 (PMC10190069; doi:10.1186/s41065-023-00280-1)
Supplement: Supplementary file 1 — Additional file 1: Supplementary Table 1. EZH2 shRNA sequences. Supplementary Table 2. RT-qPCR primer sequences. Supplementary Table 3. The list of EZH2 overexpression and knockdown stably transfected cell line. Supplementary Table 4. The list of Top 30 DEGs. Supplementary Figure 1. Western blot detected the expression level of EZH2 protein in the establishment of EZH2 overexpression and knockdown cell models. A, C and E showed the BXPC-3 cells, B, D and F showed the HPDE6-C7 cells. The internal reference target was β-actin gene. *,**,*** represented significant differences between the different of EZH2 stable transfected cells models, P < 0.05, P < 0.01, and P < 0.001, respectively. Supplementary Figure 2. Effect of EZH2 on cell proliferation detected by colony formation experiment. A and C showed the BXPC-3 cells, B and D showed the HPDE6-C7 cells. Supplementary Figure 3. Gating strategy for living/dead cells exclusion by staining 7AAD. Supplementary Figure 4. Ki-67 antibody experiments tested the effect of EZH2 on cell proliferation. A and C showed the BXPC-3 cells, B and D showed the HPDE6-C7 cells. *,**,*** represented significant differences between the different of EZH2 stable transfected cells models, P < 0.05, P < 0.01, and P < 0.001, respectively. Supplementary Figure 5. The influence of EZH2 on cell migration in HPDE6-C7 cell lines was detected by scratch test. *,**,*** represented significant differences between the different of EZH2 stable transfected cells models, P < 0.05, P < 0.01, and P < 0.001, respectively. Supplementary Figure 6. Scratch test detected the cell migration of EZH2 overexpression and knockdown BXPC-3 cell models. *,**,*** represented significant differences between the different of EZH2 stable transfected cells models, P < 0.05, P < 0.01, and P < 0.001, respectively. [file 41065_2023_280_MOESM1_ESM.doc]

Supplementary Figure 1


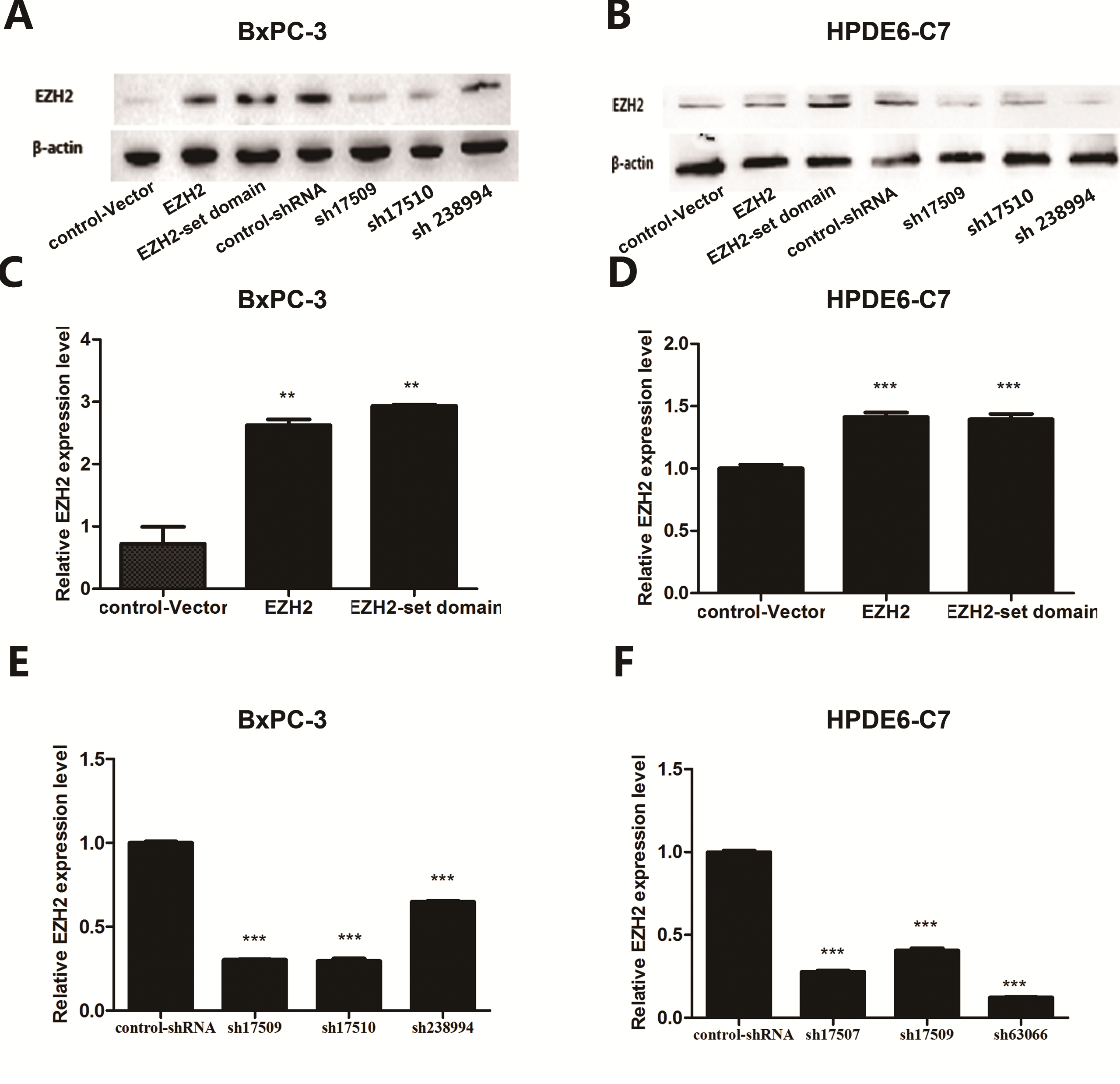


Supplementary Figure 2


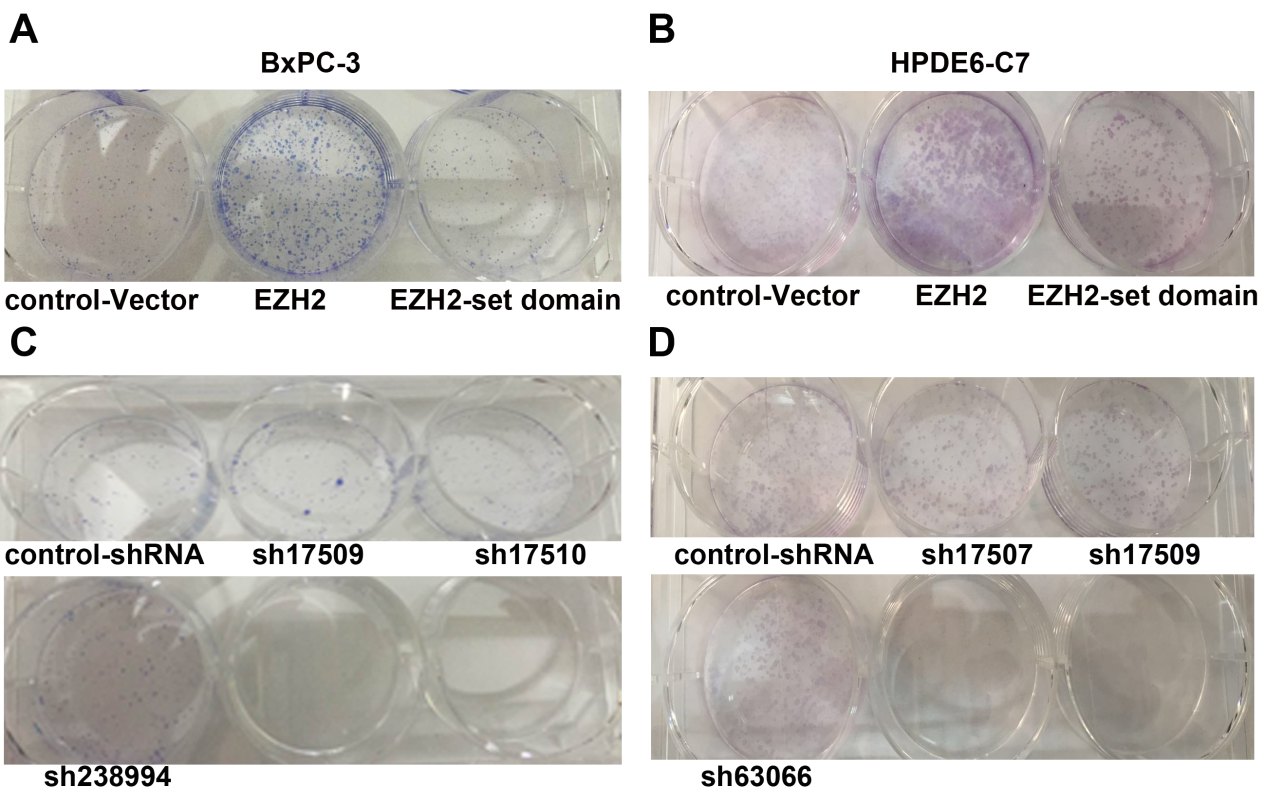


Supplementary Figure 3


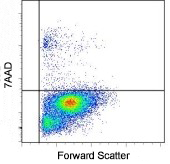


Supplementary Figure 4


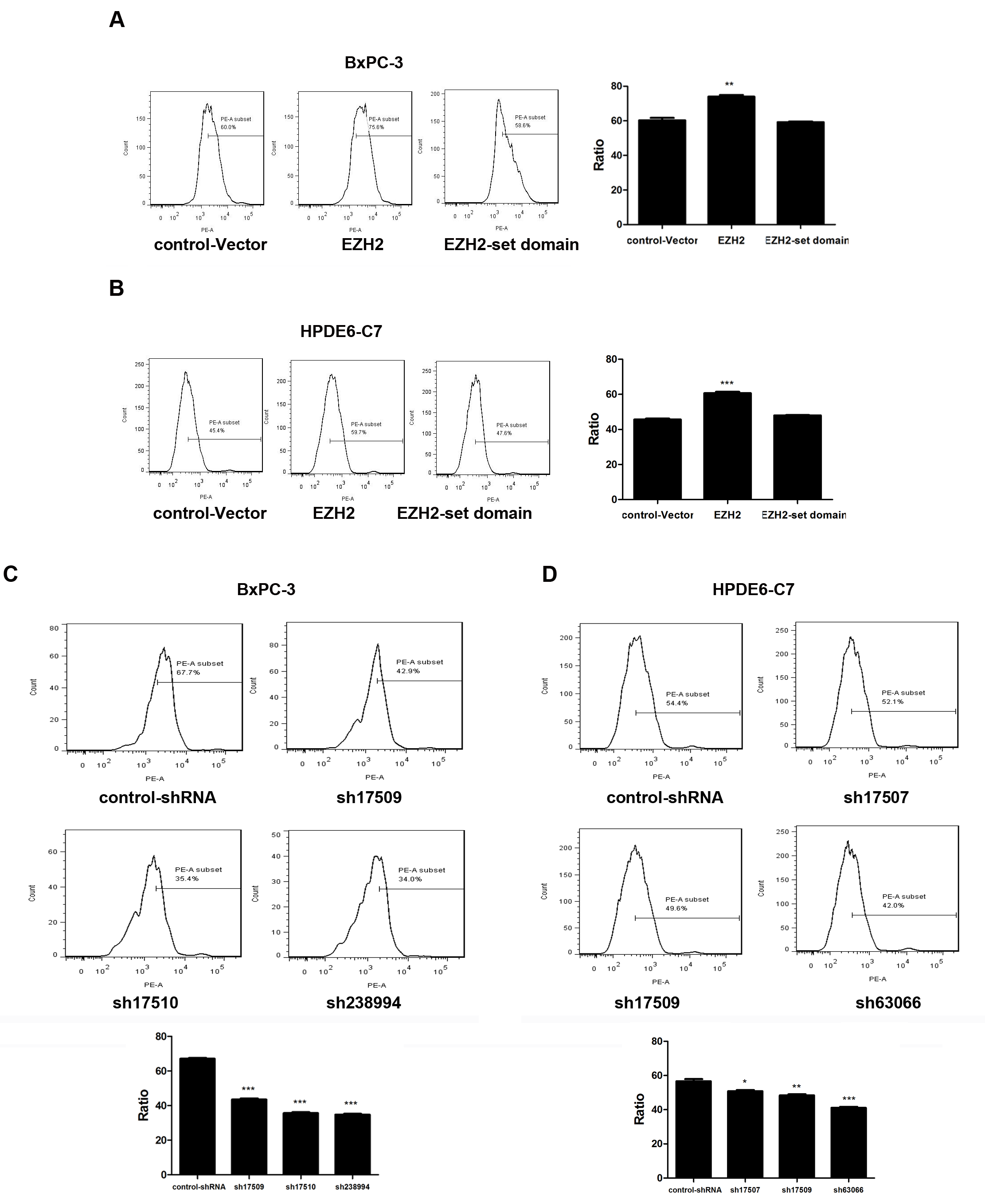


Supplementary Figure 5


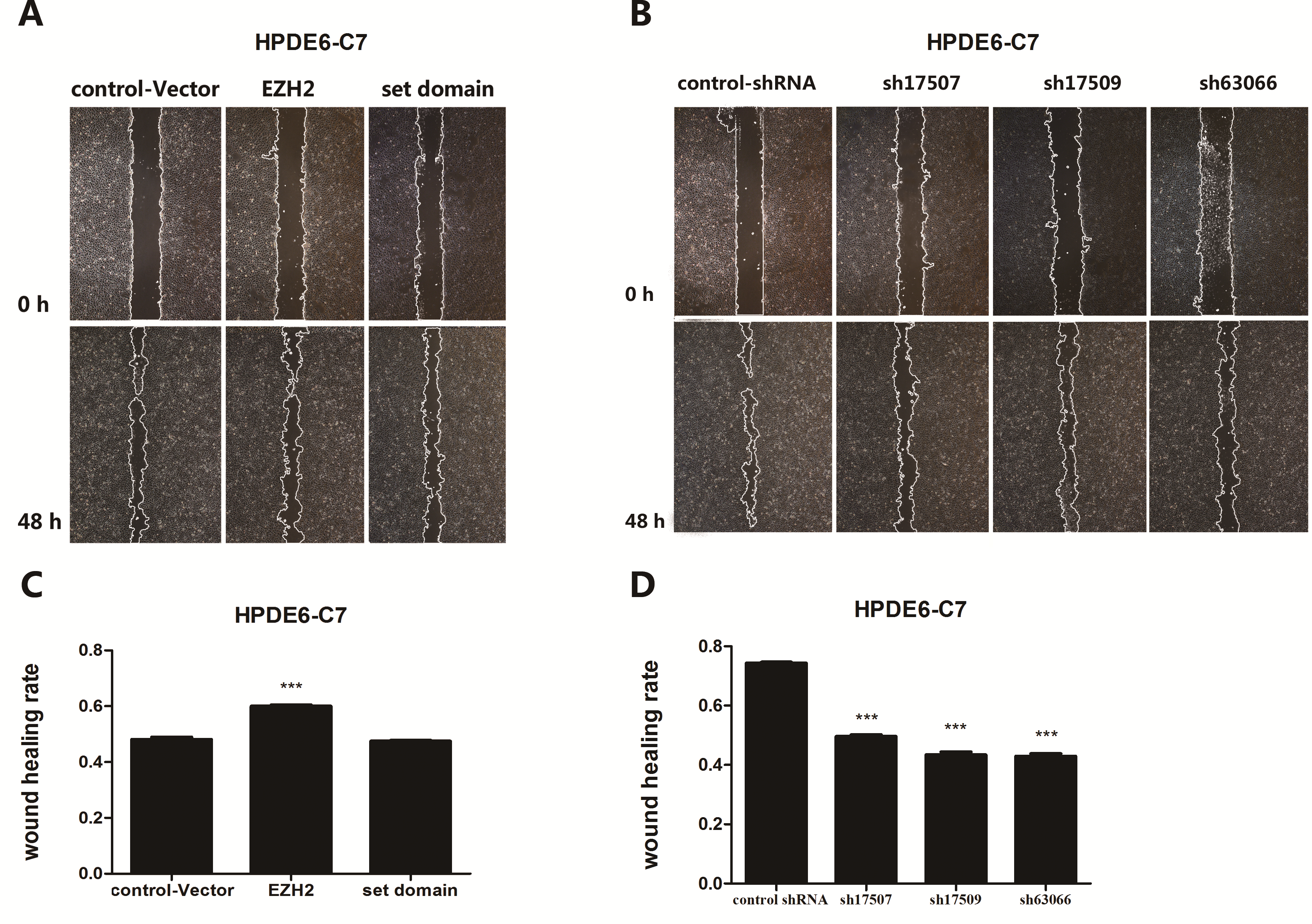


Supplementary Figure 6


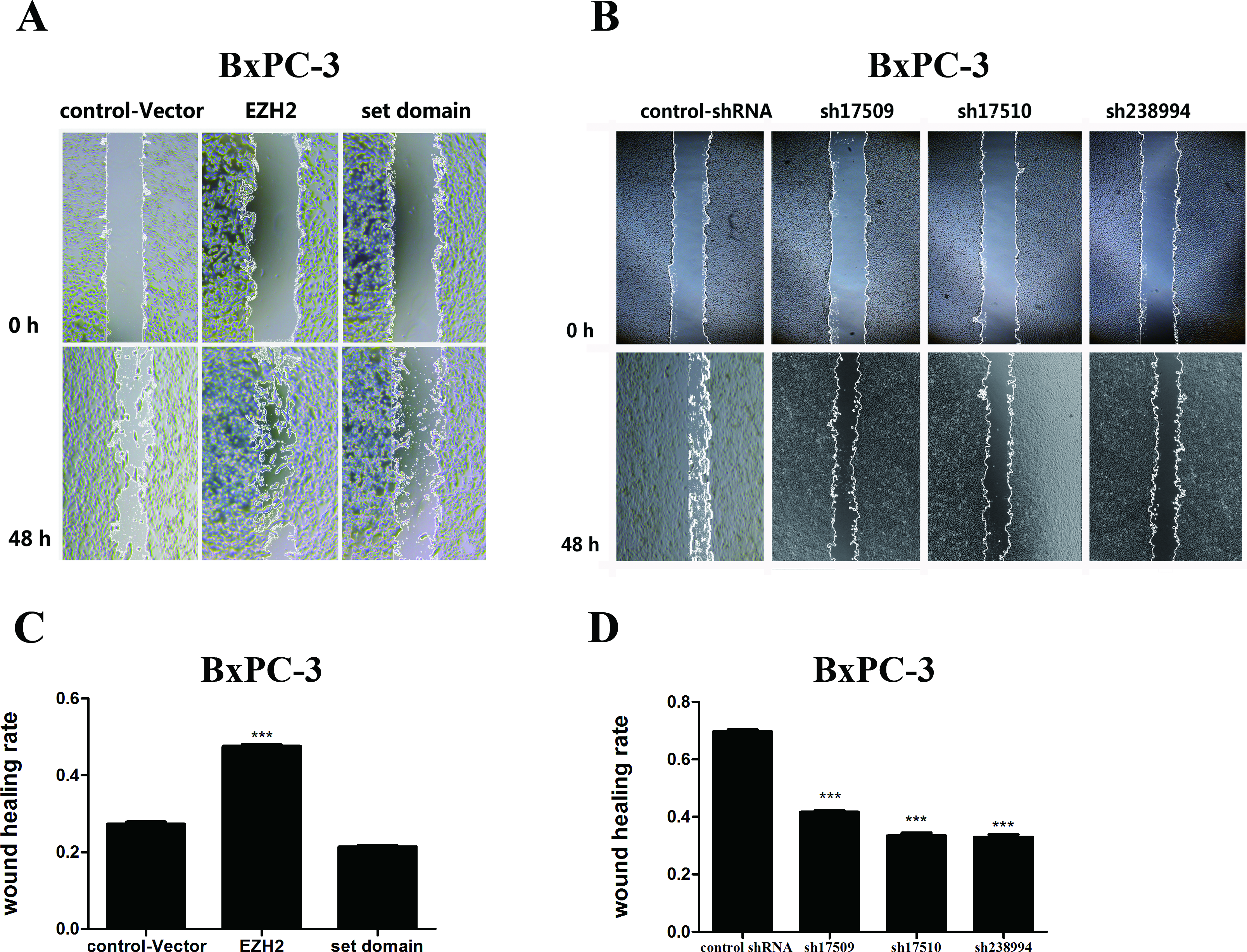


Supplementary table 1 EZH2 shRNA sequences

| Name | Number | Sequence (5’-3’) |
| --- | --- | --- |
| sh 17507 | V2LHS_17507 | TGTGCTATCACACAAGGGC |
| sh 17509 | V2LHS_17509 | CATCTTGAGAAATAATCTC |
| sh 17510 | V2LHS_17510 | TTATCATACACTTTCCCTC |
| sh 238994 | V2LHS_238994 | TTAAGATTTCCGTTCTTTC |
| sh 63066 | V2LHS_63066 | TTACTGTCCCAATGGTCAG |

Supplementary table 2 RT-qPCR primer sequences

| Name | Sequence (5’-3’) |
| --- | --- |
| EZH2 | Forward：TACTTGTGGAGCCGCTGAC |
|  | Reverse：CTGCCACGTCAGATGGTG |
| E2F1 | Forward：GCTCTCCGAGGACACTGACAG |
|  | Reverse：ATCCGGGACAACAGCGGT |
| GLI1 | Forward：CCAGTCATCCTGCAGCAGTGA |
|  | Reverse：GGCTGACAGTATAGGCAGAGCTG |
| CDK3 | Forward：AAGAGATTGTGCCCAATCTGGA |
|  | Reverse：AGGGCAGTCTTGGCTGTGAT |
| Mcm4 | Forward：CGGAAAGAAGAATTAGCTGAAGCAT |
|  | Reverse：CAGGGCACGCAGTGCTTC |
| β-actin | Forward：CTGGACTTCGAGCAAGAGAT |
|  | Reverse：GATGTCCACGTCACACTTCA |

Supplementary table 3 The list of EZH2 overexpression and knockdown stably transfected cell line

| The name of stably transfected cell | HPDE6-C7 cell | BxPC-3 cell |
| --- | --- | --- |
| control-vector | CD510-B-1 control-vector | CD510-B-1 control-vector |
| EZH2 overexpression | CD510-B-1-EZH2 | CD510-B-1-EZH2 |
| EZH2 set domain deletion | CD510-B-1-EZH2-set1 | CD510-B-1-EZH2-set1 |
| control-shRNA | pGIPZ control-shRNA | pGIPZ control-shRNA |
| EZH2 knockdown | EZH2-sh17507 | EZH2-sh17509 |
|  | EZH2-sh17509 | EZH2-sh17510 |
|  | EZH2-sh63066 | EZH2-sh238994 |

Supplementary table 4 The list of Top 30 DEGs

| Gene name | chromosomal location | log2FC | Differential expression |
| --- | --- | --- | --- |
| CTA-384D8.31 | chr22:50984843-50986081 | 10.811 | Up |
| HMSD | chr18:61616534-61672278 | 10.041 | Up |
| RN7SK | chr6:52860417-52860748 | 9.609 | Up |
| RP11-68I3.2 | chr17:27887564-28513493 | 9.581 | Up |
| CTD-2571L23.8 | chr19:48110780-48246391 | 8.098 | Up |
| RP11-20E24.1 | chr12:76956073-77009211 | 6.957 | Up |
| TPTE2P5 | chr13:41396431-41495910 | 6.922 | Up |
| GUSBP4 | chr6:58246050-58256569 | 6.740 | Up |
| RP11-495P10.6 | chr1:147744013-147752855 | 6.324 | Up |
| RP11-867G23.8 | chr11:66115420-66139685 | 5.736 | Up |
| RP11-445H22.4 | chr20:43285091-43379675 | 5.064 | Up |
| BCO2 | chr11:111895537-112095422 | 4.786 | Up |
| ANO7P1 | chr1:16542403-16554522 | 4.510 | Up |
| RP6-206I17.1 | chr1:143647637-143745417 | 4.425 | Up |
| USP12-AS2 | chr13:27746395-27757135 | 4.142 | Up |
| PSKH1 | chr16:67927174-67970990 | 3.986 | Up |
| CDKL1 | chr14:50704280-50883179 | -3.859 | Down |
| CTA-292E10.6 | chr22:29196670-29244547 | -3.940 | Down |
| AC020594.5 | chr2:33661390-33789817 | -4.058 | Down |
| RP11-692N5.1 | chr18:9615261-9619361 | -4.088 | Down |
| NANOS3 | chr19:13972876-13991571 | -4.122 | Down |
| C11orf95 | chr11:63527363-63536113 | -4.169 | Down |
| MIPEPP3 | chr13:21872277-21922860 | -4.570 | Down |
| BVES-AS1 | chr6:105544696-105627870 | -5.016 | Down |
| CTD-2192J16.22 | chr19:12754088-12792716 | -5.050 | Down |
| CTD-2245F17.3 | chr19:53700363-53714268 | -5.219 | Down |
| AC073043.1 | chr2:200732118-200775882 | -5.551 | Down |
| DPH6-AS1 | chr15:35838395-36151202 | -7.615 | Down |
| AC068499.10 | chr19:18315539-18366229 | -7.820 | Down |
| ZNF137P | chr19:53091757-53193749 | -8.014 | Down |

DEGs: differentially expressed genes; FC: fold change.
